# Discovery of a Novel Acetylcholinesterase Inhibitor by Fragment-Based Design and Virtual Screening

**DOI:** 10.3390/molecules26072058

**Published:** 2021-04-03

**Authors:** Georgi Stavrakov, Irena Philipova, Atanas Lukarski, Mariyana Atanasova, Borislav Georgiev, Teodora Atanasova, Spiro Konstantinov, Irini Doytchinova

**Affiliations:** 1 Department of Chemistry, Faculty of Pharmacy, Medical University of Sofia, Sofia 1000, Bulgaria; alukarski@pharmfac.mu-sofia.bg (A.L.); matanasova@pharmfac.mu-sofia.bg (M.A.); tatanasova@pharmfac.mu-sofia.bg (T.A.); skonstantinov@pharmfac.mu-sofia.bg (S.K.); 2 Institute of Organic Chemistry with Centre of Phytochemistry, Bulgarian Academy of Sciences, Sofia 1113, Bulgaria; irena@orgchm.bas.bg; 3 Institute of Biodiversity and Ecosystem Research, Bulgarian Academy of Sciences, Sofia 1113, Bulgaria; bobogeorgiev5@gmail.com

**Keywords:** acetylcholinesterase inhibitor, fragment-based library, molecular docking, BBB permeability, galantamine, neurotoxicity, neuro-2A cell line, Ellman’s method

## Abstract

Despite extensive and intensive research efforts in recent decades, there is still no effective treatment for neurodegenerative diseases. On this background, the use of drugs inhibiting the enzyme acetylcholinesterase (AChE) remains an eternal evergreen in the symptomatic treatment of mild to moderate cognitive impairments. Even more, the cholinergic hypothesis, somewhat forgotten in recent years due to the shift in focus on amyloid cascade, is back to life, and the search for new, more effective AChE inhibitors continues. We generated a fragment-based library containing aromatic moieties and linkers originating from a set of novel AChE inhibitors. We used this library to design 1220 galantamine (GAL) derivatives following the model GAL (binding core) - linker (L) - aromatic fragment (Ar). The newly designed compounds were screened virtually for blood–brain barrier (BBB) permeability and binding to AChE. Among the top 10 best-scored compounds, a representative lead molecule was selected and tested for anti-AChE activity and neurotoxicity. It was found that the selected compound was a powerful non-toxic AChE inhibitor, 68 times more active than GAL, and could serve as a lead molecule for further optimization and development.

## 1. Introduction

Acetylcholinesterase (AChE) is one of the most active enzymes in the central cholinergic system [1]. At the cholinergic synapses, it catalyzes the degradation of the neurotransmitter acetylcholine (ACh) to choline and acetyl and terminates its action on the postsynaptic ACh receptors. An extensive network of cortical pyramidal neurons, rich in AChE, has been observed by histochemical analysis of human brains [2]. It has been found that these neurons are absent at birth and during childhood, gradually emerge during adolescence, reach a maximum in early adulthood, and then gradually decrease in the course of normal aging. In patients with neurodegenerative disorders, a severe loss of cholinergic neurons in the cortex and hippocampus is observed due to abnormal accumulation of misfolded proteins like amyloid-β (Aβ), τ-protein, α-synuclein [3,4,5,6,7]. This severe loss is one of the factors responsible for cognitive impairments, typical for Alzheimer’s and Parkinson’s diseases [8,9].

In this regard, the use of drugs able to inhibit the enzyme AChE and hence, to increase the levels of ACh in the brain and to improve the cognitive functions has a clear, rational benefit in the treatment of neurodegenerative disorders especially Alzheimer’s disease (AD). Currently, the AChE inhibitors like donepezil, galantamine and rivastigmine, are the only FDA- and EMA-approved drugs for the symptomatic treatment of mild to moderate cognitive impairments [10]. The levels of AChE inhibition found in patients treated by current AChE inhibitors are less than the minimum of 50% required for effective therapy of AD [11]. Any attempt to increase the dose to achieve a better AChE inhibition results in an increase in drug toxicity and limits the clinical benefits [12]. Thus, the demand for more powerful and less toxic AChE inhibitors is still an extremely important and urgent scientific challenge.

Galantamine (GAL) is one of the most widely prescribed drugs for treating AD [13]. Apart from being an AChE inhibitor, GAL is an allosteric modulator of nicotinic ACh receptor [14], has antioxidant properties [15] and facilitates the clearance of Aβ peptide in rat brains [16]. Due to this variety of actions, GAL can serve as а multitarget scaffold to design new derivatives with a higher AChE inhibitory effect and lower toxicity. Steadily in recent decades, GAL is the focus of many lab projects around the world aiming to develop safer and more powerful anti-neurodegenerative agents [17,18,19,20,21,22,23,24,25,26,27].

**Figure 1:**
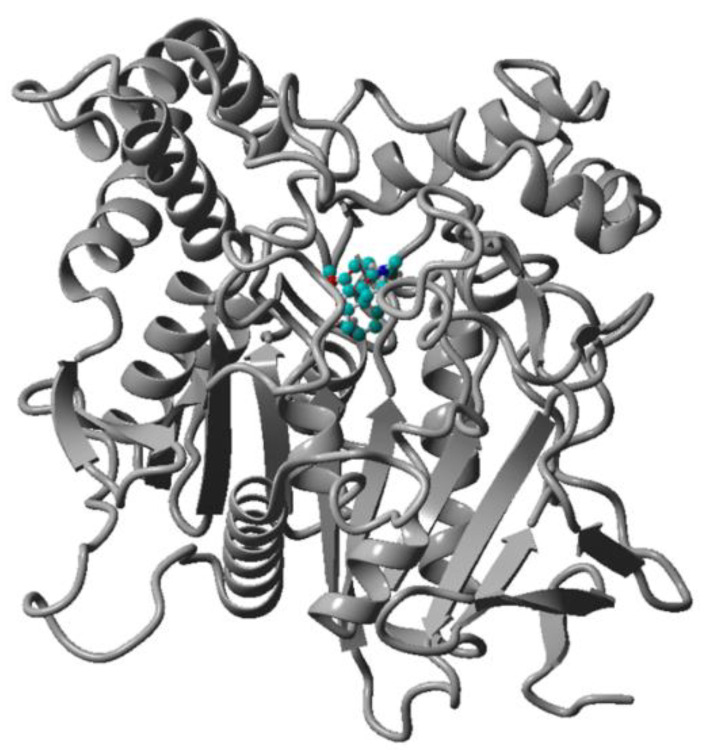
X-ray structure of the complex galantamine (GAL)-*rh*AChE (pdb code: 4ey6). Protein is given in gray ribbon, GAL—in ball-and-stick colored by element.

In the present study, we use GAL as a core structure and design a series of derivatives to improve the binding affinity to AChE. Several well-defined domains exist in the deep and narrow binding site of AChE. Among them of greatest importance is the active catalytic site (CAS), consisting of the triad Ser203, Glu334 and His447, and responsible for ACh’s hydrolysis [28]. Along the binding gorge are situated the anionic, acyclic, and oxyanion domains. Near the gorge entrance is placed the peripheral anionic site (PAS), which is associated with the initiation of amyloidogenesis [29]. The X-ray structure of the complex *rh*AChE-GAL [28] (Figure 1) shows that GAL binds at the bottom of the binding site where the CAS is located and fills only one-third of the binding gorge. The GAL ammonium group is pointing towards the entrance of the binding gorge and is the only position in the GAL molecule available for substitution without affecting the binding mode.

**Figure 2:**
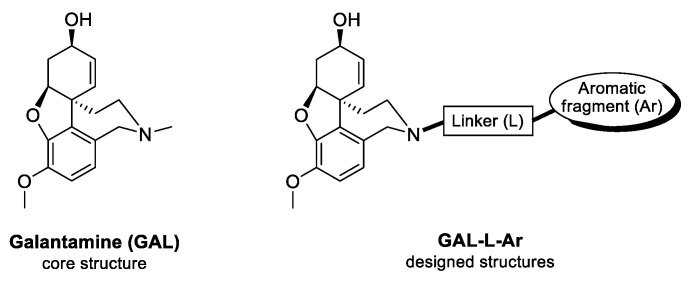
Common structure of the designed galantamine derivatives (GAL-L-Ar).

Our previous structure—AChE affinity studies on GAL derivatives showed that the substitution of CH_3_ group at the quaternary N-atom with a linker containing five to seven carbon atoms and ending with aromatic moiety increases the affinity to AChE more than 1000 times [24]. Here, we designed a fragment-based library containing aromatic moieties and linkers originating from a set of novel AChE inhibitors recently discovered in our lab by docking-based virtual screening of a ZINC lead-like database [30]. We used this library to design GAL derivatives following the model GAL-linker (L)-aromatic fragment (Ar) (Figure 2), screened them virtually for blood–brain barrier (BBB) permeability and docked them into *rh*AChE. Among the best-scored inhibitors, a representative lead molecule was selected and tested for anti-AChE activity and neurotoxicity.

## 2. Results

### 2.1. Design of GAL Derivatives Using Fragment-Based Libraries

**Figure 3:**
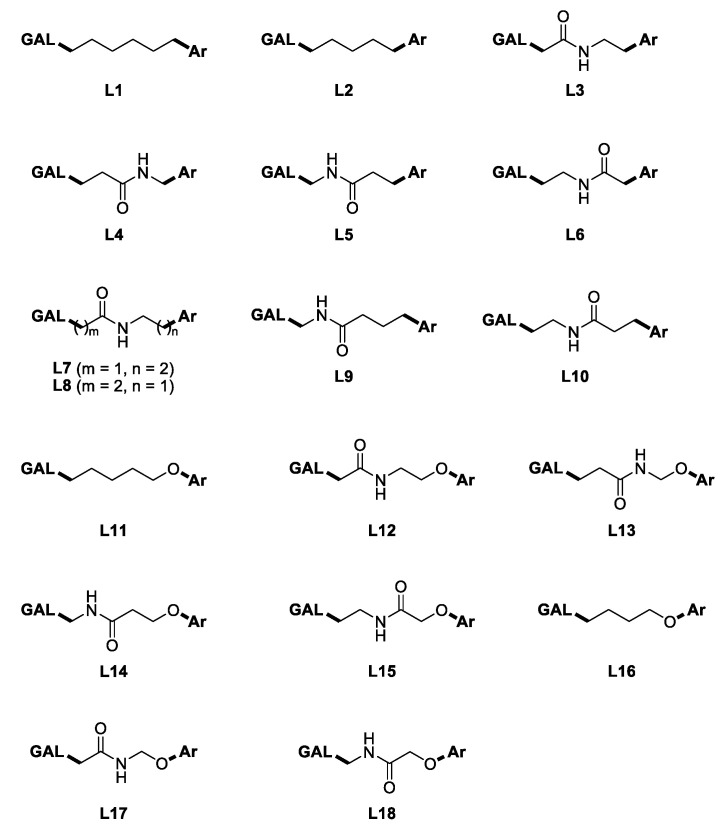
Linkers used in the design of ed galantamine hybrids.

The novel GAL derivatives were designed to contain a core, a linker, and an aromatic group (Figure 2). The GAL molecule was used as a core. The linkers were designed following our previous structure–activity studies on GAL derivatives [22,23,24,25,26,27], showing that a linker containing five to seven carbon atoms is optimal for dual-site binding to AChE. This finding was applied to select linkers from the AChE inhibitors discovered recently from the ZINC database [30]. Thus, 18 types of linkers were designed and assigned by L1–18 (Figure 3).

**Figure 4:**
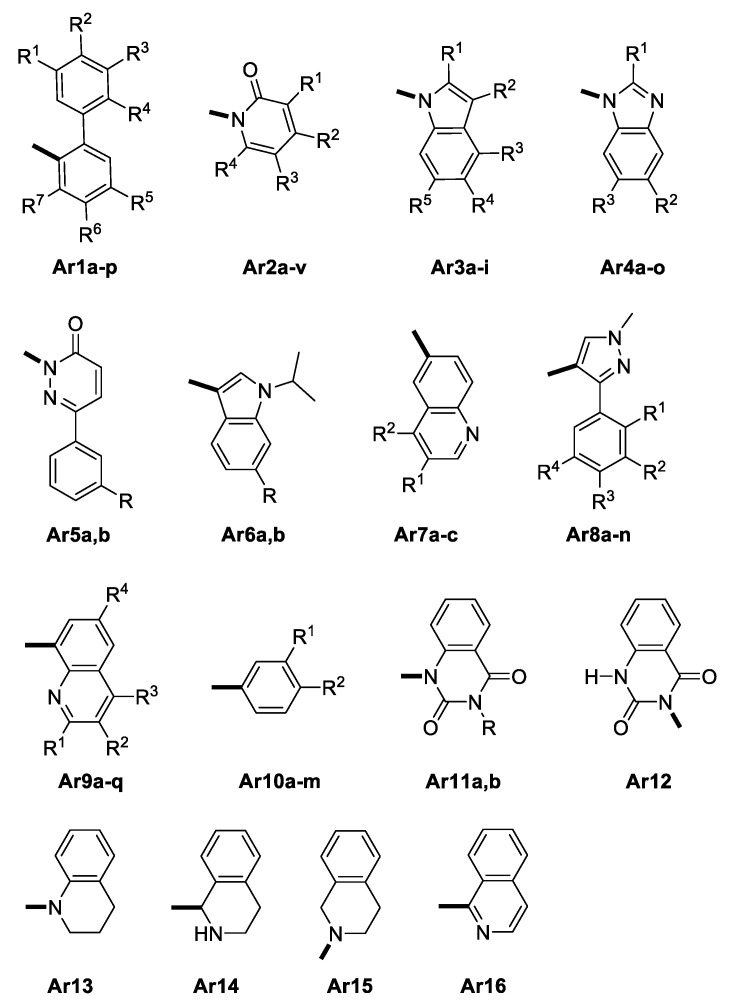
Structures of the aromatic fragments. The full fragment library is given in Table S1.

Sixteen aromatic groups (assigned by Ar1–16) were designed to mimic the aromatic rings of ZINC AChE inhibitors (Figure 4) [30]. Each group consists of derivatives (denoted by letters a–v) of the corresponding aromatic core or a single aromatic moiety (groups Ar12–16). The following aromatic fragments were used in the design of the novel set of GAL derivatives: biphenyl (Ar1), pyridine-2(1*H*)-one (Ar2), 1*H*-indole (Ar3), 1*H*-benzo[*d*]imidazole (Ar4), 6-phenylpyridazin-3(2*H*)-one (Ar5), 1-isopropyl-1*H*-indole (Ar6), quinoline (Ar7, Ar9), 1-methyl-3-phenyl-1*H*-pyrazole (Ar8), phenyl (Ar10), quinazoline-2,4(1*H*,3*H*)-dione (Ar11, Ar12), 1,2,3,4-tetrahydroquinoline (Ar13), 1,2,3,4-tetrahydroisoquinoline (Ar14, Ar15) and isoquinoline (Ar16). The substituents vary between F, Cl, Br, NH_2_, methyl, methoxy, *i*-propyl, CHF_2_, SO_2_NH_2_, NHSO_2_CH_3_, NHSO_2_NH_2_. Thus, 120 aromatic moieties were designed (16 of Ar1, 22 of Ar2, 9 of Ar3, 15 of Ar4, 2 of Ar5, Ar6 and Ar11, 3 of Ar7, 14 of Ar8, 17 of Ar9, 13 of Ar10 and 1 of Ar12–16). For each aromatic fragment, ten linkers (L1–10) were used, except for Ar13–16, where seven linkers (L1–3, L6–8, and L10) were used. For Ar9d, Ar9e, Ar9h and Ar9j, eight linkers (L11–L18) were used additionally. In total, 1220 novel GAL derivatives were designed in the present study: 160 with Ar1, 220 with Ar2, 90 with Ar3, 150 with Ar4, 20 with Ar5, 20 with Ar6, 30 with Ar7, 140 with Ar8, 202 with Ar9, 130 with Ar10, 20 with Ar11, 10 with Ar12, 7 with Ar13, 7 with Ar14, 7 with Ar15, 7 with Ar16. The full fragment library used in the present study is given in Table S1 from Supplementary Materials.

### 2.2. Blood–Brain Barrier (BBB) Permeability

The 1220 newly designed GAL derivatives were tested for BBB permeability applying the filters implemented in SwissADME [31] and BBB Predictor [32]. SwissADME has one BBB permeability filter, and 381 compounds passed it. Then, these compounds were filtered through the 8 algorithms of the BBB predictor, and 199 passed all of them. Their distribution by groups was as follows: 22 with Ar1, 25 with Ar2, 17 with Ar3, 20 with Ar4, 10 with Ar5, 3 with Ar6, 4 with Ar7, 21 with Ar8, 39 with Ar9, 38 with Ar10, and none with Ar11–16.

### 2.3. Virtual Screening by Molecular Docking

The 199 molecules that passed the nine BBB permeability filters were docked to human rhAChE. GAL was used as a referent structure. Eight compounds with RMSD >1.5 Å were excluded. The GoldScores of the remaining 191 derivatives ranged from 115.38 to 77.40, and all of them were higher than the GoldScore of GAL (77.73), except for one (77.40). One hundred sixty-six compounds (87%) had scores below 100, while 25 compounds (13%) showed scores above 100 (Table 1).

### 2.4. GAL Derivative Selected for Synthesis

Nine of the top 10 best-scored GAL derivatives contained the aromatic fragment Ar1, and seven of them-the linker L10. Among them, compound **8** includes the basic structure of Ar1, i.e., Ar1a, and is a good representative lead of both Ar1 and L10. This compound was selected for further synthesis and tests.

The physicochemical properties of compound 8 are given in Table 2. It has almost twice the molecular weight of GAL, lower *pK_a_*, higher *logP* and *logD_7.4_*, wider polar surface area (*PSA*), 2 hydrogen-bond donors and 6 acceptors. The HBDs is the enol OH group of GAL core and the linker’s NH group. All heteroatoms in the molecule act as HBAs.

### 2.5. Synthesis of the Selected GAL Derivative

**Scheme 1:**
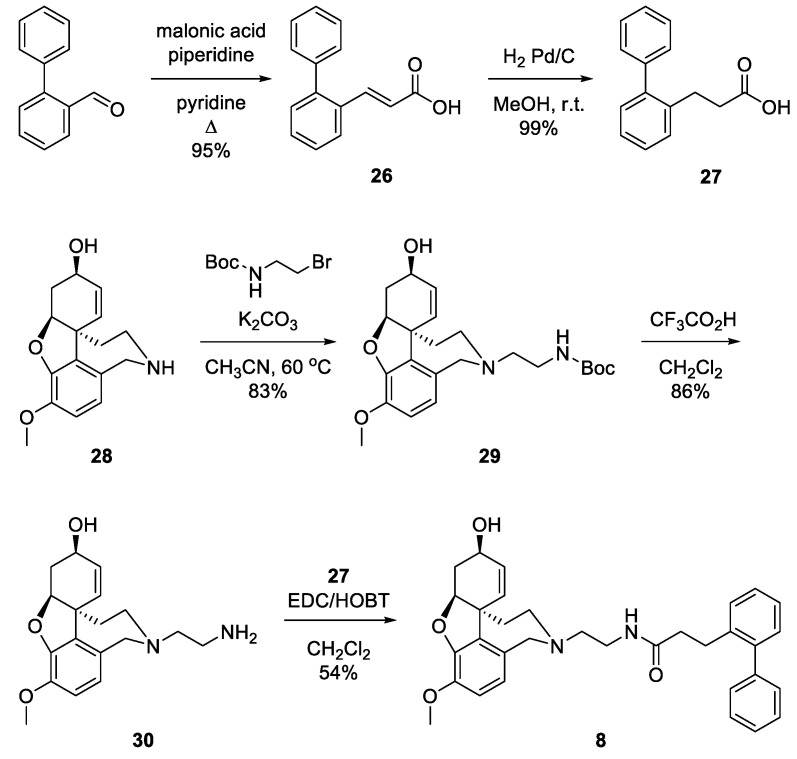
Synthesis of the selected lead compound **8**.

Two building blocks-an an acid **27** and an amine **30,** were needed for the synthesis of compound 8, which were subsequently connected via an amide bond. Thus, initial *Knoevenagel* condensation of biphenyl-2-carbaldehyde with malonic acid afforded the unsaturated acid **26**, which was hydrogenated to give 3-(biphenyl-2-yl)propanoic acid **27**. Nucleophilic substitution of *tert*-butyl *N*-(2-bromoethyl)carbamate with norgalantamine **28** gave the the *tert*-butylcarbamate protected diamine **29**. The latter was deprotected and immediately subjected to amide bond formation with acid **27** to give the selected compound **8** in a moderate yield (Scheme 1).

### 2.6. Neurotoxicity of the Selected GAL Derivative

The cytotoxicity of compound **8** was tested on neuro-2A cells as described in the Materials and Мethods section. No toxicity was observed in concentrations up to 100 µM (Table 2). For comparison, the toxicity of GAL on neuro-2A is >50 µM [24]. Thus, the newly designed GAL derivative is practically non-toxic.

### 2.7. Anti-AChE Activity of the Selected GAL Derivative

The inhibitory activity of compound **8** was measured in vitro on the enzyme acetylcholinesterase from electric eel (eeAChE) by Ellman’s method [33] with some modifications [34]. However, the docking simulations were conducted on human AChE (*rh*AChE). The sequence alignment of the two proteins has shown that the 17 residues forming the deep and narrow binding gorge on the enzyme are identical [25]. Hence, the in vitro test on *ee*AChE assesses adequately the inhibitory activities of compounds generated from molecular docking simulations on *rh*AChE, as it was proven in several studies [22,23,24,25,26,27].

The selected compound **8** inhibited the enzyme AChE with IC_50_ = 27.79 nM (Table 2). It was 68 times more active than GAL, which showed IC_50_ = 1.92 µM in the same test.

### 2.8. Stability of the Complex AChE-Compound **8** assessed by Molecular Dynamics Simulation

The best docking pose of compound **8** into *rh*AChE was selected as starting coordinates of the complex for the molecular dynamics (MD) simulations. The complex was solvated in saline in an octahedral box, energy minimized, heated to 310 °C, equilibrated and simulated for 1000 ns (1µs) by AMBER v. 18 (UCSF, San Francisco, US) [35]. For the time of simulation, the complex was stable, and the ligand remained bound in the binding site.

## 3. Discussion

**Figure 5:**
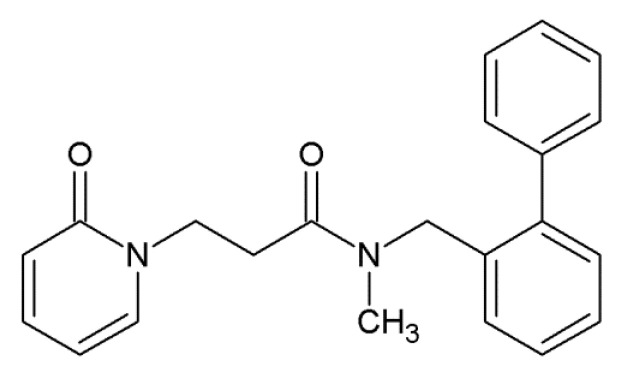
One of the most active novel AChE inhibitors derived previously by a docking-based screening of a ZINC lead-like database with K_d_ = 0.525 µM, measured by isothermal titration calorimetry [30].

GAL is a safe but moderate inhibitor of AChE. To improve its inhibitory activity, in the present study, we designed a library of fragments generated from compounds discovered previously as AChE inhibitors [30]. The library consisted of two sections: aromatic fragments and linkers. Sixteen aromatic fragments with various substituents and 18 linkers were derived and combined with being attached to GAL as a main binding core. Thus, a fragment-based library of 1220 GAL derivatives was generated. Initially, the compounds were screened for the ability to cross the BBB. One hundred ninety-nine derivatives (16%) passed the nine BBB filters of SwissADME and BBB Predictor. Next, these compounds were docked into the *rh*AChE and assessed by the GoldScore function of GOLD v.5.2.2 (CCDC, Cambridge, UK). Among the top 10 of the best-scored derivatives, 9 contained the aromatic fragment Ar1, and 7 contained the linker L10. The fragment Ar1 and the linker L10 originate from one of the most active novel AChE inhibitors (Figure 5) derived by a docking-based screening of a ZINC lead-like database containing 6,053,287 molecules [30].

Compound **8** was selected as a representative lead molecule of GAL derivatives with Ar1 and L10. It was synthesized and tested for cytotoxicity and AChE activity. It was found that this derivative is non-toxic and inhibits the enzyme with IC_50_ of 28 nM, which makes it 68 times more active than GAL. The enzyme–inhibitor complex is stable for 1 µs MD simulation in saline at 310 K. These initial tests on compound **8** make it a perspective lead of a new series of congeners with potential AChE inhibitory activity.

**Figure 6:**
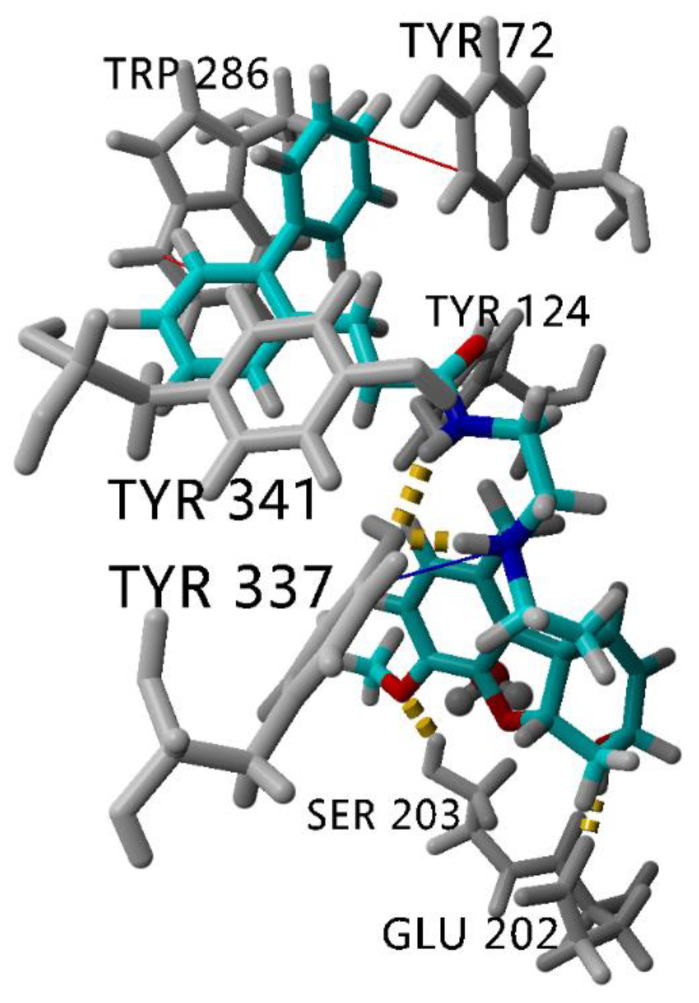
The best-scored docking pose of compound **8** into *rh*AChE. Hydrogen bonds are shown in yellow dashes, cation-π interaction-in blue line and π-π interaction-in red line. The enzyme residues are given as gray sticks. Compound **8** is colored by element. The structural water molecule within the binding site is presented in ball-and-stick. Interactions are visualized by YASARA v. 17.1.28 (IMBM, Graz, Austria) [36].

The best-scored docking pose of compound **8** and its interactions with the enzyme are visualized in Figure 6. The GAL core is located on the bottom of the binding gorge and forms a network of hydrogen bonds: between GAL’s quaternary ammonium and Tyr337, between OCH_3_ and Ser203, and between OH and Glu202. A cation-π interaction is formed between the positively charged ammonium atom and Tyr337. An additional hydrogen bond is formed between the linker’s NH group and Tyr337. The aromatic fragment is involved in a π-π stacking with Tyr72 and Trp286. Similar interactions were observed between the other top-scored GAL derivatives (data not shown). For compounds **2** and **10**, the linker’s carbonyl O atom makes a hydrogen bond with Tyr124. In the complexes of compounds **3**, **4**, **6**, and **7**, the aromatic fragments form π-π stacking with Tyr124 instead of Tyr72, while in the complex of compound **10,** this stacking is formed with Tyr341.

## 4. Materials and Methods

### 4.1. Materials

Reagents were commercial grade and used without further purification. Thin-layer chromatography (TLC) was performed on aluminum sheets pre-coated with Merck Kieselgel 60 F254 0.25 mm (Merck, Sofia, Bulgaria). Flash column chromatography was carried out using Silica Gel 60 230–400 mesh (Fluka Labimex, Sofia, Bulgaria). Commercially available solvents were used for reactions, TLC and column chromatography. Melting points were determined in capillary tubes on SRS MPA100 OptiMelt (Sunnyvale, CA, USA) automated melting point system with the heating rate set at 1°C/min (uncorrected). Optical rotation ([α]^20^_D_) was measured on JASCO *P-*2010 POLARIMETER (JASCO INTERNATIONAL CO. Tokyo, Japan). The NMR spectra were recorded on a Bruker Avance II+ 600 (BRUKER BioSpin GmbH, Rheinstetten, Germany) (600.13 for ^1^H-MHz and 150.92 MHz for ^13^C-NMR) spectrometer with TMS as internal standards for chemical shifts (δ, ppm). ^1^H- and ^13^C-NMR data are reported as follows: chemical shift, multiplicity (s = singlet, d = doublet, t = triplet, q = quartet, br = broad, m = multiplet), coupling constants (Hz), integration, identification. The assignment of the ^1^H- and ^13^C-NMR spectra were made based on COSY and HSQC experiments. Elemental analyses were performed by the Microanalytical service Laboratory of the Institute of Organic Chemistry, Bulgarian Academy of Science.

### 4.2. Synthesis and Analytical Data of (E)-3-(biphenyl-2-yl)acrylic acid **26**

A mixture of biphenyl-2-carbaldehyde (0.300 g, 1.65 mmol), malonic acid (0.343 g, 3.29 mmol) and a catalytic amount of piperidine was refluxed overnight in 2 mL dry pyridine. The mixture was cooled and acidified with 1 N HCl. The resulting mixture was extracted three times with ether. Then the organic phase was extracted with aq. Na_2_CO_3_. Then aqueous phase was acidified with 1 N NCl and extracted with ether. The combined organic extracts were dried over MgSO_4_, filtered and concentrated under reduced pressure. The residue was purified by flash column chromatography (silica gel, petroleum ether/EtOAc = 1:2) to give 0.352 g of **26** as white crystals, m.p. 201–202 °C. Yield: 95%. ^1^H-NMR (CDCl_3_, 600 MHz): δ = 11.70 (br, 1H, OH), 7.81 (d, *J* = 15.9 Hz, 1H, 3-H), 7.72 (dt, *J* = 8.3, 0.5 Hz, 1H, arom.), 7.48–7.43 (m, 3H, arom.), 7.42–7.38 (m, 3H, arom.), 7.32–7.30 (m, 2H, arom.), 6.39 (d, *J* = 15.9 Hz, 1H, 2-H) ppm. ^13^C-NMR (CDCl3, 150.9 MHz): δ = 171.97 (CO), 146.14 (C-3), 143.20 (C, arom.), 139.67 (C, arom.), 132.15 (C, arom.), 130.57 (CH, arom.), 130.27 (CH, arom.), 129.80 (2CH, arom.), 128.33 (2CH, arom.), 127.69 (CH, arom.), 127.97 (CH, arom.), 126.97 (CH, arom.), 118.08 (C-2) ppm.


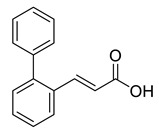


### 4.3. Synthesis and Analytical Data of 3-(biphenyl-2-yl)propanoic acid **27**

To a solution of **26** (0.300 g, 1.34 mmol) in MeOH (10 mL), flashed with Ar and cooled to 0 °C, 10% Pd/C was added at one portion, and the atmosphere was changed to H_2_ with the help of a hydrogen-filled balloon. The mixture was monitored by TLC, and after 2 h at room temperature, no traces of the starting compound were detected. The mixture was filtered through a pad of Celite and concentrated to give 0.300 g of **27** as white crystals, m.p. 112–113 °C. Yield: 99%. ^1^H-NMR (CDCl_3_, 600 MHz): δ = 11.77 (br, 1H, OH), 7.42–7.40 (m, 2H, arom.), 7.37–7.34 (m, 1H, arom.), 7.31–7.27 (m, 5H, arom.), 7.22–7.21 (m, 1H, arom.), 2.93 (t, *J* = 7.8 Hz, 1H, 3-H), 2.45 (t, *J* = 8.2 Hz, 1H, 2-H) ppm. ^13^C-NMR (CDCl_3_, 150.9 MHz): δ = 178.6 (CO), 141.99 (C, arom.), 141.32 (C, arom.), 137.46 (C, arom.), 130.27 (CH, arom.), 129.01 (2CH, arom.), 128.91 (CH, arom.), 128.25 (2CH, arom.), 127.61 (CH, arom.), 127.05 (CH, arom.), 126.31 (CH, arom.), 34.89 (C-2), 28.00 (C-3) ppm.


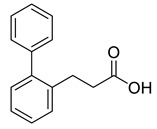


### 4.4. Synthesis and Analytical Data of tert-butyl {2-[(4aS,6R,8aS)-6-hydroxy-3-methoxy- 5,6,9,10-tetrahydro-4aH-[1]benzofuro[3a,3,2-ef][2]benzazepin-11(12H)-yl]ethyl}carbamate **29**

To a solution of norgalanthamine **28** (0.100 g, 0.36 mmol) in anhydrous acetonitrile (10 mL) under argon atmosphere was added tert-butyl *N*-(2-bromoethyl)carbamate (0.098 g, 0.44 mmol) and anhydrous K_2_CO_3_ (0.150 g, 1.08 mmol). After stirring for 24 h at 60 °C, the mixture was filtered through a pad of Celite. The filtrate was concentrated under reduced pressure and subjected to purification by flash column chromatography on silica gel (CH_2_Cl_2_/CH_3_OH/NH_4_OH = 25/1/0.02) to give the desired product **29** as waxy solid. Yield: 83%. [α]_D_^20^ = −64.5 (c 0.4205, CHCl_3_). ^1^H-NMR (CDCl_3_, 600 MHz): δ = 6.65 (d, *J* = 8.2 Hz, 1H, H-2), 6.61 (d, *J* = 8.2 Hz, 1H, H-1), 6.06 (d, *J* = 10.2 Hz, 1H, H-8), 6.01 (dd, *J* = 10.2, 4.9 Hz, 1H, H-7), 5.04 (br, 1H, NH), 4.60 (br, 1H, H-6), 4.15 (d, *J* = 15.6 Hz, 1H, H-12), 4.14–4.12 (m, 1H, H-4a), 3.83 (s, 3H, OCH_3_), 3.78 (d, *J* = 15.6 Hz, 1H, H-12), 3.40 (t, *J* = 13.7 Hz, 1H, H-10), 3.20–3.18 (m, 2H, H-1′), 3.14 (d, *J* = 14.5 Hz, 1H, H-10), 2.70–2.66 (m, 1H, H-5), 2.65–2.59 (m, 2H, H-2′), 2.40 (br, 1H, OH), 2.04–1.98 (m, 2H, H-5, H-9), 1.54–1.51 (m, 1H, H-9), 1.44 (s, 9H, C(CH_3_)_3_) ppm. ^13^C-NMR (CDCl_3_, 150.9 MHz) δ = 156.05 (CO), 145.83 (C-3a), 144.23 (C-3), 133.01 (C-12b), 127.75 (C-7, C-12a), 126.62 (C-8), 122.13 (C-1), 111.10 (C-2), 88.67 (C-6), 79.19 (C(CH3)3), 62.00 (C-4a), 56.98 (C-12), 55.85 (OCH3), 51.65 (C-10), 49.60 (C-2′),48.38 (C-8a), 37.30 (C-1′), 32.85 (C-9), 29.87 (C-5), 28.40 (C(CH_3_)_3_) ppm.


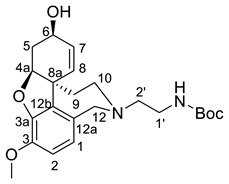


### 4.5. Synthesis and Analytical Data of 3-(biphenyl-2-yl)-N-{2-[(4aS,6R,8aS)-6-hydroxy- 3-methoxy-5,6,9,10-tetrahydro-4aH-[1]benzofuro[3a,3,2-ef][2]benzazepin-11(12H)-yl]ethyl}propanamide **8**

To a solution of **29** (0.100 g, 0.24 mmol) in dichloromethane (5 mL) was added CF_3_CO_2_H (0.25 mL) dropwise at 0 °C. The mixture was stirred for 2 h at r.t. and quenched by dropwise addition of aq NH_4_OH. The mixture was extracted with CH_2_Cl_2_. The organic layers dried and concentrated to give 0.125 g of crude (4a*S*,6*R*,8a*S*)-11-(2-aminoethyl)-3-methoxy-5,6,9,10,11,12-hexahydro-4a*H*-[1]benzofuro[3a,3,2-ef][2]benzazepin-6-ol **30**, which was used without further purification. Yield: 86%.

To a solution of acid **27** (0.052 g, 0.23 mmol) in CH_2_Cl_2_ (7 mL) was added EDC (0.044 g, 0.23 mmol), HOBT (0.031 g, 0.23 mmol) and amine **30** (0.065 g, 0.21 mmol). The mixture was stirred for 1 h at r.t. and the product formation was monitored by TLC. The mixture was concentrated to dry and directly subjected to flash column chromatography on silica gel (CH_2_Cl_2_/MeOH/NH_4_OH = 20/1/0.02) to give 0.058 g of the desired product 8 as white amorphous solid, m.p. 131–133 °C. Yield: 54%. [α]_D_^20^ = -56.7 (c 0.4115, CHCl_3_). ^1^H-NMR (CDCl_3_, 600 MHz): δ = 7.42–7.40 (m, 2H, arom.), 7.36–7.34 (m, 1H, arom.), 7.32–7.28 (m, 3H, arom.), 7.27–7.24 (m, 2H, arom.), 7.22–7.21 (m, 1H, arom.), 6.66 (d, *J* = 8.2 Hz, 1H, H-2), 6.58 (d, *J* = 8.2 Hz, 1H, H-1), 6.06 (dd, *J* = 10.4, 0.8 Hz, 1H, H-8), 6.02 (dd, *J* = 10.2, 4.7 Hz, 1H, H-7), 5.78 (br, 1H, NH), 4.60 (br, 1H, H-6), 4.15 (br, 1H, H-4a), 4.09 (d, J = 15.6 Hz, 1H, H-12), 3.83 (s, 3H, OCH_3_), 3.65–3.63 (m, 1H, H-12), 3.63 (t, *J* = 13.8 Hz, 1H, H-10), 3.24–3.14 (m, 2H, CH_2_NHCO), 3.03 (d, *J* = 14.7 Hz, 1H, H-10), 2.97 (t, *J* = 7.8 Hz, 2H, COCH_2_), 2.71–2.68 (m, 1H, H-5), 2.56–2.55 (m, 1H, NCH_2_), 2.50–2.46 (m, 1H, NCH_2_), 2.25 (t, *J* = 8.0 Hz, 2H, COCH_2_CH_2_), 2.02–1.94 (m, 2H, H-5, H-9), 1.51 (d, *J* = 12.6 Hz, 1H, H-9) ppm. ^13^C-NMR (CDCl_3_, 150.9 MHz) δ = 172.21 (CO), 145.83 (C-3a), 144.28 (C-3), 141.79 (C, arom.), 141.52 (C, arom.), 138.09 (C, arom.), 132.93 (C-12b), 130.18 (CH, arom.), 129.26 (CH, arom., C-12a), 129.02 (2CH, arom.), 128.23 (2CH, arom.), 127.83 (C-7), 127.53 (CH, arom.), 126.95 (CH, arom.), 126.50 (C-8), 126.20 (CH, arom.), 122.11 (C-1), 111.12 (C-2), 88.64 (C-6), 61.96 (C-4a), 56.81 (C-12), 55.85 (OCH3), 51.54 (C-10), 48.92 (NCH_2_), 48.34 (C-8a), 37.48 (COCH_2_CH_2_), 35.95 (CH_2_NHCO), 32.79 (C-9), 29.86 (C-5), 29.22 (COCH_2_) ppm. C_33_H_36_N_2_O_4_ (524.65): calcd. C 75.55, H 6.92, N 5.34, found C 75.23, H 7.09, N 5.06.


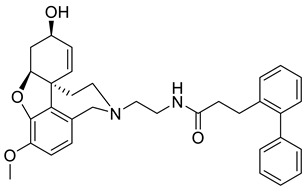


### 4.6. Prediction of Blood–Brain Barrier (BBB) Permeability

The ability of the GAL derivatives to cross the BBB by passive diffusion was predicted by SwissADME (http://www.swissadme.ch Accessed on: 5 February 2019) [31] and BBB Predictor (https://www.cbligand.org/BBB/predictor.php Accessed on: 5 February 2019 ) [32]. SwissADME predicts BBB permeability based on the lipophilicity and polarity of small molecules [37]. The BBB predictor uses a support vector machine (SVM) and LiCABEDS (ligand classifier of adaptively boosting ensemble decision stumps) algorithms developed based on four types of fingerprints for 1593 compounds with known BBB permeability [38].

### 4.7. Virtual Screening by Molecular Docking

The GAL derivatives were constructed with Avogadro software v. 1.1.0 (University of Pittsburgh, Pittsburgh, PA, USA) [39] and minimized with MMFF94s force field [40], then structures were docked into the X-ray structure of human recombinant acetylcholinesterase (*rh*AChE, pdb ID: 4EY6, R = 2.40 Å) [28]. The docking simulations were performed by GOLD v.5.2.2 (CCDC Ltd., Cambridge, UK) using a protocol previously optimized in terms of scoring function, rigid/flexible ligand and binding site, the radius of the binding site, presence/absence of a water molecule (HOH846) within the binding site, number of genetic algorithms (GA) runs [22,23,24,25,26,27]. The docking simulations in the present study were performed at the following settings: scoring function GoldScore, flexible ligand, flexible binding site, the radius of the binding site 10 Å, a structural water molecule within the binding sire (HOH 846), 100 GA runs. The residues from the binding site, which are in close proximity to the bound ligand, were set as flexible. These were Tyr72, Asp74, Trp86, Tyr124, Ser125, Trp286, Phe297, Tyr337, Phe338, Tyr341. GAL from X-ray structure was used as a referent molecule. The best-scored compounds were analyzed for synthetic feasibility and protein–ligand interactions.

### 4.8. Neurotoxicity Test

To assess the neurocytotoxicity of the selected compound, murine neuroblastoma neuro-2A cells (German collection DSMZ, Braunschweig, Germany) were used. They were cultivated under standard conditions: complete medium (90% DMEM, 10% heat-inactivated FBS, and non-essential amino acids); 37 °C and 5% CO_2_ in a fully humidified atmosphere. The cell line was kept in the logarithmic growth phase by splitting 1:4 once a week using trypsin/EDTA. About 30% of the cells grew like neuronal cells. For the experimental evaluation of the cytotoxicity, the cells were plated in 96-well flat-bottomed plates at the recommended density of around 1100 cells/25 cm^2^. After 24 h, the cells were treated with a series of concentrations (100, 50, 25, 12.5, 6.25 µM) of the tested compound, and after 72 h incubation, an MTT-dye reduction assay was performed [41]. MTT stock solution (10 mg/mL in PBS) was added (0.01 mL/well) at the end of incubation. Plates were further incubated at 37 °C for 4 h. Next, the formazan crystals were dissolved by the addition of 0.110 mL/well 5% formic acid in 2-propanol (*v/v*). Absorption was measured at 580 nm wavelength on an automated ELISA reader Labexim LMR1. At least six wells per concentration were used, and data were processed using the software 2.0 (GraphPad, San Diego, CA, USA).

### 4.9. Assessment of AChE Inhibitory Activity

The microplate assay used for measuring AChE inhibitory activity was as described by Ellman [33] with the modifications added by López [34]. Acetylthiocholine iodide (ATCI) in a solution with 5,5′-dithiobis(2-nitrobenzoic acid) (DTNB) was used as a substrate for the acetylcholinesterase from *Electrophorus electricus* (Steinheim, Sigma-Aldrich, Germany). 50 μL of AChE (0.25 U/mL) dissolved in phosphate buffer (8 mM K_2_HPO_4_, 2.3 mM NaH_2_PO_4_, 0.15 M NaCl, pH 7.5) and 50 μL of the sample dissolved in the same buffer were added to the wells. The plates were incubated for 30 min at room temperature before the addition of 100 μL of the substrate solution (0.04 M Na_2_HPO_4_, 0.2 mM DTNB, 0.24 mM ATCI, pH 7.5). The absorbances were read in a microplate reader (BIOBASE, ELISA-EL10A, Jinan, Shandong, China) at 405 nm after 3 min. Enzyme activity was calculated as an inhibition percentage compared to an assay using a buffer without any inhibitor. GAL was used as a positive control. The AChE inhibitory data were analyzed with the software package Prism 9 (GraphPad Inc., San Diego, USA). The IC_50_ values were measured in triplicate, and the results are presented as means ± SD.

### 4.10. Molecular Dynamics Protocol

The best-scored pose of the selected GAL derivative in complex with AChE was used as a starting structure for the MD simulation by Amber 18 [35]. The small molecule was parametrized using GAFF2.11 force field and AM1-BCC charges, and the complex was solvated in a truncated octahedral box with TIP3P water and 0.15 M NaCl. The system was subjected to energy minimization, heating to 310 K, density equilibration, preproduction equilibration and production dynamics for 1µs (1000 ns). Frames were saved every 1 ns.

## 5. Conclusions

The fragment-based design of 1220 GAL derivatives and their virtual screening for BBB permeability and docking into *rh*AChE delivered a set of 25 novel prospective AChE inhibitors. One of them was selected as a representative of the most common linker and aromatic fragment among the top 10 best-scored ligands. The in vitro tests for cytotoxicity and AChE inhibitory activity confirmed that the selected compound is a powerful non-toxic AChE inhibitor and can serve as a lead molecule for further optimization and development.

## Figures and Tables

**Table 1:** The best-scored GAL derivatives with GoldScore >100. Compound **8** (given in bold) was selected for synthesis.

Compound No.	GoldScore	Linker	Aromatic Fragment
**1**	115.38	L10	Ar1d
**2**	114.14	L10	Ar1h
**3**	111.49	L10	Ar1n
**4**	111.28	L10	Ar1m
**5**	110.42	L5	Ar1p
**6**	109.51	L10	Ar1i
**7**	108.14	L10	Ar1o
**8**	**105.56**	**L10**	**Ar1a**
**9**	105.53	L6	Ar1a
**10**	103.90	L6	Ar10e
**11**	103.73	L1	Ar8l
**12**	103.24	L2	Ar4e
**13**	102.87	L10	Ar1e
**14**	102.87	L7	Ar13
**15**	102.84	L6	Ar1j
**16**	102.49	L2	Ar8n
**17**	102.31	L2	Ar8e
**18**	102.08	L6	Ar1k
**19**	101.87	L10	Ar6a
**20**	101.87	L3	Ar10e
**21**	101.67	L3	Ar10l
**22**	101.03	L6	Ar1b
**23**	100.60	L1	Ar9o
**24**	100.31	L1	Ar3d
**25**	100.04	L1	Ar15
**GAL**	77.73		

**Table 2:** Physicochemical properties, neurotoxicity and anti-AChE activity of compound **8**.

Parameter	Compound 8	GAL
*MW* ^1^	524.65	287.35
*pKa*	7.10	7.92
*logP*	4.49	1.75
*logD*_7.4_	4.34	1.12
Polar surface area *(Å)*	71.03	41.93
Hydrogen-bond donors *(HBD)*	2	1
Hydrogen-bond acceptors *(HBA)*	6	4
Neurotoxicity on neuro-2A cells (*IC*_50_)	>100 µM	>50 µM ^2^
Inhibitory activity on *ee*AChE (*IC*_50_)	27.79 ± 5.49 nM	1.92 ± 0.1 µM

^1^ The physicochemical properties were calculated by ACD/logD v.9.08 (ACD Inc., Toronto, Canada). ^2^ [24].

## Data Availability

Not applicable.

## Supplementary Materials

The following are available online, Table S1: Fragment library used in the present study.

## Sample Availability

Sample of the compound **8** is available from the authors.
