# Supplementary material for: Discovery of a Novel Acetylcholinesterase Inhibitor by Fragment-Based Design and Virtual Screening
Source: Molecules. 2021 Apr 3;26(7):2058. doi: 10.3390/molecules26072058 (PMC8038331; doi:10.3390/molecules26072058)
Supplement: Supplementary file 1 [file molecules-26-02058-s001.pdf]

Table S1. Fragment library used in the present study.

Aromatic fragment	Type	R^1	R^2	R^3	R^4	R^5	R^6	R^7
Ar1	**a**	H	H	H	H	H	H	H
	**b**	H	H	H	Cl	H	H	H
	**c**	Cl	Cl	H	H	H	H	H
	**d**	CH_3	H	H	H	H	H	H
	**e**	H	F	H	H	H	H	H
	**f**	H	H	H	H	H	Cl	H
	**g**	Cl	F	H	H	H	H	H
	**h**	H	H	H	CH_3	H	H	H
	**i**	H	H	H	F	H	H	H
	**j**	H	H	Br	H	H	H	H
	**k**	H	Br	H	H	H	H	H
	**l**	H	H	H	OCH_3	H	H	H
	**m**	H	H	H	H	H	H	F
	**n**	H	H	H	H	H	F	H
	**o**	H	H	H	H	F	H	H
	**p**	F	H	H	H	H	H	H
Ar2	**a**	H	H	H	H	-	-	-
	**b**	NH_2	H	H	H	-	-	-
	**c**	H	NH_2	H	H	-	-	-
	**d**	CH_3	H	H	H	-	-	-
	**e**	H	CH_3	H	H	-	-	-
	**f**	H	H	CH_3	H	-	-	-
	**g**	H	H	H	CH_3	-	-	-
	**h**	F	H	H	H	-	-	-
	**i**	H	F	H	H	-	-	-
	**j**	H	H	F	H	-	-	-
	**k**	H	H	H	F	-	-	-
	**l**	Cl	H	H	H	-	-	-
	**m**	H	Cl	H	H	-	-	-
	**n**	H	H	Cl	H	-	-	-
	**o**	H	H	H	Cl	-	-	-
	**p**	Br	H	H	H	-	-	-
	**q**	H	Br	H	H	-	-	-
	**r**	H	H	Br	H	-	-	-
	**s**	H	H	H	Br	-	-	-
	**t**	H	OCH_3	H	H	-	-	-
	**u**	H	H	OCH_3	H	-	-	-
	**v**	OCH_3	H	H	H	-	-	-
Ar3	**a**	H	H	H	H	H	-	-
	**b**	CH_3	H	H	H	H	-	-
	**c**	H	CH_3	H	H	H	-	-
	**d**	H	F	H	H	H	-	-

	**e**	H	H	H	Br	H	-	-
	**f**	H	CH_3	H	Br	H	-	-
	**g**	H	H	Br	H	H	-	-
	**h**	H	H	H	OCH_3	H	-	-
	**i**	H	H	H	OCH_3	OCH_3	-	-
**Ar4**	**a**	H	H	H	-	-	-	-
	**b**	H	H	Br	-	-	-	-
	**c**	H	H	CH_3	-	-	-	-
	**d**	CH_3	H	H	-	-	-	-
	**e**	H	Br	H	-	-	-	-
	**f**	H	CH_3	CH_3	-	-	-	-
	**g**	H	H	OCH_3	-	-	-	-
	**h**	H	H	Cl	-	-	-	-
	**i**	H	OCH_3	OCH_3	-	-	-	-
	**j**	*i*C_3H_7	H	H	-	-	-	-
	**k**	CHF_2	H	H	-	-	-	-
	**l**	H	Cl	Cl	-	-	-	-
	**m**	H	Cl	H	-	-	-	-
	**n**	H	OCH_3	H	-	-	-	-
	**o**	H	CH_3	H	-	-	-	-
**Ar5**	**a**	H	-	-	-	-	-	-
	**b**	Br	-	-	-	-	-	-
**Ar6**	**a**	H	-	-	-	-	-	-
	**b**	Br	-	-	-	-	-	-
**Ar7**	**a**	H	H	-	-	-	-	-
	**b**	Br	H	-	-	-	-	-
	**c**	H	Br	-	-	-	-	-
**Ar8**	**a**	H	H	H	H	-	-	-
	**b**	F	H	H	H	-	-	-
	**c**	H	F	H	H	-	-	-
	**d**	H	H	F	H	-	-	-
	**e**	CH_3	H	H	H	-	-	-
	**f**	Cl	H	H	H	-	-	-
	**g**	H	Cl	H	H	-	-	-
	**h**	H	H	Cl	H	-	-	-
	**i**	H	Br	H	H	-	-	-
	**j**	H	H	Br	H	-	-	-
	**k**	H	OCH_3	H	H	-	-	-
	**l**	H	H	OCH_3	H	-	-	-
	**m**	H	F	H	F	-	-	-
	**n**	H	$C\text{-}R^2 = N$	H	H	-	-	-
**Ar9**	**a**	H	H	H	H	-	-	-
	**b**	H	Br	H	H	-	-	-
	**c**	H	H	Br	H	-	-	-
	**d**	Cl	H	H	H	-	-	-
	**e**	H	H	Cl	H	-	-	-

	**f**	H	F	H	H	-	-	-
	**g**	H	H	F	H	-	-	-
	**h**	CH_3	H	H	H	-	-	-
	**i**	H	CH_3	H	H	-	-	-
	**j**	OCH_3	H	H	H	-	-	-
	**k**	H	OCH_3	H	H	-	-	-
	**l**	H	H	H	F	-	-	-
	**m**	H	H	H	Br	-	-	-
	**n**	H	H	H	OCH_3	-	-	-
	**o**	H	H	H	CH_3	-	-	-
	**p**	H	H	OCH_3	H	-	-	-
	**q**	H	H	CH_3	H	-	-	-
**Ar10**	**a**	OCH_3	H	-	-	-	-	-
	**b**	H	OCH_3	-	-	-	-	-
	**c**	OCH_3	OCH_3	-	-	-	-	-
	**d**	F	H	-	-	-	-	-
	**e**	H	F	-	-	-	-	-
	**f**	Br	H	-	-	-	-	-
	**g**	H	Br	-	-	-	-	-
	**h**	H	SO_2NH_2	-	-	-	-	-
	**i**	H	$NHSO_2CH_3$	-	-	-	-	-
	**j**	H	$NHSO_2NH_2$	-	-	-	-	-
	**k**	Cl	H	-	-	-	-	-
	**l**	H	Cl	-	-	-	-	-
	**m**	Cl	Cl	-	-	-	-	-
**Ar11**	**a**	H	-	-	-	-	-	-
	**b**	CH_3	-	-	-	-	-	-
